# Attitudes toward Vaccination against COVID-19 in Poland. A Longitudinal Study Performed before and Two Months after the Commencement of the Population Vaccination Programme in Poland

**DOI:** 10.3390/vaccines9050503

**Published:** 2021-05-13

**Authors:** Mateusz Babicki, Agnieszka Mastalerz-Migas

**Affiliations:** Department of Family Medicine, Wroclaw Medical University, 51-141 Wroclaw, Poland; agnieszka.mastalerz-migas@umed.wroc.pl

**Keywords:** COVID-19 vaccination, attitudes toward vaccination, COVID-19

## Abstract

Despite the fact that more than a year has passed since the WHO declared the pandemic, there is still no effectivetreatment of COVID-19. According to current knowledge, the only method to stop the virus from spreading is prophylactic vaccination of the population. However, to achieve herd immunity, 60–72% of the population needs to be vaccinated, which is a significant challenge for current healthcare systems. As it has already been proven, having an effective vaccine is not the same as using it. Public acceptance is essential here. The study aimed to assess the changes in the attitudes of online respondents toward vaccination against COVID-19 over time. In the research, a questionnaire designed by the author of the study was used and it was distributed via the Internet in two stages. The questionnaire included a section assessing a sociodemographic status as well as the questions, designed by the author, evaluating the willingness of the respondents to get vaccinated and their main concerns associated with it. The first stage covered the period before the commencement of the population vaccination programme in Poland, i.e., 14–27 December 2020. Then, the survey was redistributed 2 months after the vaccination programme was started, i.e., 1–19 March 2021. Participation in the study was fully anonymous, voluntary and there was a possibility to opt out at any stage of the research. A total of 2048 respondents were surveyed and 26 persons refused to be involved in the research. A total number of 2022 responses were analysed. Stage I of the research involved 22.9% of the respondents (463 persons). The questions were answered by 1559 persons (72.9%) after the survey was redistributed. Among the participants of stage II of the study, 422 (27.1%) persons have already been vaccinated against COVID-19. A comparison of the responses that were collected from both stages of the study showed a slight increase in the willingness to get vaccinated against COVID-19 over time. It turned out that women, residents of large cities, people with a higher level of education and healthcare workers showed a more favourable attitude toward vaccination. According to the results of the survey, chronic diseases do not have a significant impact on the attitude toward vaccinations (*p* = 0.155). As the social vaccination promotion campaign continued, a slight increase in the willingness to get vaccinated was observed. According to the survey, women, residents of large cities and those with higher education demonstrated more favourable attitudes toward getting vaccinated against COVID-19. It should be stressed that despite the passage of time and the increasing experience with the new types of vaccines against COVID-19, the percentage of people who are afraid of the complications after the vaccination has not decreased significantly and the concern related to the ineffectiveness of vaccination has dramatically increased. This demonstrates the limited effectiveness of the current information system regarding passing the knowledge on of the safety and efficiency of vaccination and it indicates a necessity to modernise it as soon as possible.

## 1. Introduction

Since 11 March 2021, when the WHO declared the pandemic due to the spread of SARS-CoV-2, a vast majority of the scientific world has been focused on the search for effective treatment and preventive methods against the coronavirus [1]. Attempts have been made to treat COVID-19 with the use of antiviral drugs, antibiotics, steroids, antimalarial medications, anti-parasitic drugs, and even convalescent plasma or biologic medical products were applied to fight the infection. Unfortunately, the majority of the proposed solutions do not affect the course of COVID-19 [2,3]. The situation required the implementation of appropriate preventive measures as soon as possible. For this reason, research on preventive vaccinations was taking place at the same time. As time passed, the first information regarding the effectiveness of the preparations developed by various pharmaceutical companies appeared in the world of science [4]. On 21 December 2020, more than a year after the first case of viral pneumonia was reported which was caused by COVID-19 infection, the European Medicines Agency approved, conditionally, the first COVID-19 vaccine in Europe which was developed by Pfizer BioNTech [5]. Over the following weeks, other types of COVID-19 vaccines received conditional approval: Moderna, Astra Zeneca and Johnson & Johnson [6,7,8].

Adequate supply made it possible to start mass vaccination against COVID-19, which is still the only effective way to combat SARS-CoV-2, therefore it is extremely important to continue vaccination of the population as efficiently as possible [9]. It has been estimated that vaccination of 60–72% of the population could lead to herd immunity which would significantly hinder the spread of SARS-CoV-2 [10]. However, to achieve the above, it is necessary to coordinate the actions of the government, medical professionals and, most importantly, to increase the willingness of the public to get vaccinated. As it has already been proven, having an effective vaccine is not the same as using it. Public acceptance is essential here [11]. Currently, different attitudes toward vaccination are observed and the willingness to get vaccinated varies depending on the society; that is why constant monitoring of the situation is an essential aspect to implement appropriate strategies and achieve success [12].

The study aimed to assess public attitudes toward vaccination against COVID-19, explore the concerns regarding vaccination and identify sources of information on vaccination. The following research hypotheses were proposed: (1) Willingness to get vaccinated against COVID-19 is high enough to achieve herd immunity. (2) Over time, more and more people will show a positive attitude toward getting vaccinated against COVID-19. (3) As the population vaccination programme continues, the public’s concern regarding the effectiveness and safety of the products decreases. (4) Men, individuals with a higher level of education and those who live in cities demonstrate more favourable attitudes toward vaccination. (5) The main source of information on COVID-19 vaccination is the Internet, TV and healthcare professionals. (6) Medical workers exhibit lower levels of concern and more positive attitudes toward vaccination against COVID-19.

## 2. Materials and Methods

### 2.1. Methods

The CAWI-type study (computer-assisted web interviewing) in the form of an anonymous and fully voluntary survey, designed by the author of the research, was distributed online via a social networking site. It was addressed to Polish residents aged 18 or older. There were two stages of the study. The initial distribution of the questionnaire took place before the commencement of the COVID-19 vaccination programme in Poland, over the period from 14 to 26 December 2021. The vaccination programme began on 27 December 2020, when the first vaccine was administered to a healthcare worker. Subsequently, the process of vaccination within the healthcare sector was continued [13]. Vaccination of the remaining part of the population began on 25 January 2021 and it was initially organised for senior citizens at the age of 80 or more, and other social groups were added gradually [14]. The questionnaire was redistributed between 1 and 19 March 2021, less than two months after the commencement of the vaccination process. During the period of the data collection, the following social groups could register to get vaccinated: healthcare workers, seniors at the age of 70 or over, younger individuals who suffered from chronic conditions including persons after transplantation of tissues and/or organs, mechanically ventilated or dialysed patients and persons with oncological diseases [15,16,17,18]. Until now, the number of people vaccinated with the first dose is 3,213,724 which constitutes 8.5% of the population and there are 1,769,770 persons, i.e., 4.7% of the population, who have completed the process of vaccination [19]. During the survey distribution period, Poles could not choose the preparation with which they would be vaccinated.

Before taking part in the study, the respondents were informed about the nature of the research, the methodology and the objectives. After that, informed consent was obtained from those who were willing to participate in the project. The participants could opt out of the study at any stage without disclosing a reason. The study was approved by the Bioethics Committee of the Wroclaw Medical University and it was conducted in accordance with the Declaration of Helsinki.

The questionnaire was designed by the author of the project and it included both single-choice and multiple-choice questions. In both stages of the study, the questions involved an assessment of a socio-demographic status such as age, gender, place of residence, level of education, relationship status. There was a requirement to state whether the person was a healthcare worker and disclose medical history regarding COVID-19.

The next sections of the study included questions related to attitudes toward vaccination. The respondents were asked about their vaccination history and willingness to get vaccinated against COVID-19. The following question was asked: “Are you planning to get vaccinated against COVID-19?” with possible answers: Yes, as soon as possible/Yes, but only in a few months (up to a year)/Yes, but in a year or more/I cannot make a decision/No, but I might consider it in the future/No, never. Subsequently, the respondents were asked about their concerns regarding vaccination against COVID-19.

For the second stage of the study, there were some questions included in the survey to assess whether the respondents have already been vaccinated against COVID-19, questions regarding the presence of any chronic conditions and whether vaccination against COVID-19 should be mandatory for everyone. The respondents were also asked to indicate the type of product they would like to be vaccinated with. At the end of the survey, the respondents were asked about the sources of their knowledge regarding the COVID-19 vaccination. The English-language version of the questionnaire is available in the Supplementary Materials.

### 2.2. Statistical Analysis

The statistical analysis was conducted with the use of Statistica 13.0 software, StatSoft (Cracow, Poland, StatSoft). A chi-squared test was used to determine the relations between the compared ordinal variables. The chi-squared test with Yates correction and Fisher’s exact test was also used. While determining the relations between the variables in multiway contingency tables, correspondence analysis was used as well. The significance level of α = 0.05 was assumed in all tests that evaluated the statistical significance of the differences between mean values.

## 3. Results

### 3.1. Participants

A detailed description of the study group and the stages of the research are presented in Table 1. A total of 2048 respondents took part in the survey and 26 persons refused to be involved in the study. A total of 2022 (98.7%) responses were used for the analysis. The first stage of the study was participated by 463 persons (22.9%) with 100% informed consent. In the second stage, there were 1585 participants, 26 of whom did not agree to take part in the subsequent stages of the research, therefore there were 1559 questionnaires analysed in total (77.1%).

In the repeat survey, 529 individuals reported at least one chronic condition such as cardiovascular diseases, pulmonary system diseases, neurological diseases, psychiatric diseases, oncological disorders, skin problems, endocrine system disorders, or other. A total of 107 of the respondents reported more than 1 chronic condition. 

Out of 422 people who have been vaccinated, 89.1% are women, 231 (54.1%) are healthcare professionals, and 35% suffer from chronic conditions.

### 3.2. Approach to Vaccination

A comparison of attitudes toward vaccination against COVID-19 and the main concerns regarding vaccination are presented in Table 2. In March 2021, 422 (27.1%) out of 1559 respondents were vaccinated against COVID-19. Among the remaining group of the respondents, 51.9% indicated a willingness to get vaccinated while it was 50.7% of the respondents in stage I of the study. In December 2020 and in March 2021, the main concern of the respondents were possible complications after vaccination. Over time, a reduction from 51.8% to 47.5% was noticed, however, the value was not statistically significant (*p* = 0.265). The level of concern related to proper testing of the preparation (50.8% vs. 38.1%; *p* < 0.001) and the transport/storage of the preparation (26.4% vs. 13.4%; *p* < 0.001) was significantly reduced. There was an increase in the percentage of respondents who had no concerns related to vaccination (12.5% to 28.4%; *p* < 0.001).

Out of the respondents who took part in stage II of the survey, 622 (39.9%) believed that vaccination against COVID-19 should be mandatory while 658 (42.2%) disagreed with that statement and 278 (17.9%) had no opinion on that matter.

If there would be a possibility to choose the type of vaccine, mRNA preparations have the best reputation among the respondents. Pfizer BioNTech was indicated as a first choice by 48% of the respondents while 9.6% opted for Moderna. Johnson & Johnson was chosen by 6.1% of the respondents and AstraZeneca would be an option for only 0.6% of the surveyed persons. For 35.6% of the respondents, the type of formulation did not matter. According to the survey, the main source of knowledge regarding vaccination is the Internet—78%, followed by a healthcare worker other than a medical professional—38.7%, a medical professional—38.3%, TV—26.6%, friends—19.1%, information leaflets—18.2% and other sources of information—21.1%. More than one source of information was used by 71.4% of the respondents; 0.7% of the respondents used all the above-mentioned sources of information at the same time.

### 3.3. Influencing Factors

A detailed list of demographic variables, vaccination history, disease history and attitudes toward vaccination against COVID-19 are presented in Table 3. According to the pooled analysis, excluding the vaccinated persons, women demonstrated significantly more favourable attitudes toward vaccination against COVID-19 (*p* < 0.001) similarly to elderly persons (*p* < 0.001), people living in cities of over 250,000 residents (*p* < 0.001), individuals with a higher level of education (*p* < 0.001) and healthcare workers (*p* = 0.03). The above-mentioned results were confirmed in a separate analysis and applied to stage I and stage II of the study. The answers to the questionnaire in the case of COVID-19 convalescents were not statistically different from those obtained from persons who have not suffered from COVID-19 (*p* = 0.06).

Out of all respondents who have been vaccinated, the vast majority, 59.3%, believes that vaccination should be mandatory, 18.6% do not agree with this statement while 22.1% cannot make the decision (*p* < 0.001). 

### 3.4. Analysis of Concerns Related to Vaccination against COVID-19 

Out of the respondents, 99% who took part in stage I of the survey and 94.6% of those who participated in stage II of the research reported at least one concern related to vaccination against COVID-19; at the same time, 65% of the respondents who took part in stage I of the study declared to have more than one concern; the percentage was 60% according to the redistribution of the study. The most frequently reported concern, among persons who do not want to get vaccinated, was the fact that the vaccines have not been properly tested. A detailed summary is presented in Table 4. A summary of responses of healthcare workers and persons of other professions is presented in the Supplementary Materials Table S1. 

## 4. Discussion

According to the analysis of this study, over time, there is a gradual increase in the willingness to get vaccinated against COVID-19 among online respondents who are residents of Poland, however, there is still a large percentage of persons who are afraid of the complications and of those who do not want to get vaccinated. In December 2020, 50.7% of the respondents said that they would get vaccinated. According to the repeated survey, out of the group of 1559 respondents, 27.1%, i.e., 422 persons have already been vaccinated. Regarding the rest of the respondents, every second one declared the willingness to get vaccinated and the vast majority of the group wanted to have it done as soon as it was possible. It should be noted that the trend of total aversion toward vaccination remained high, i.e., every fourth of the respondents. The concept of implementing mandatory COVID-19 vaccination was supported by 39.9% of the respondents.

To sum up, the participants of this study show a moderate willingness to get vaccinated against COVID-19. In the literature of the subject, various attitudes regarding willingness to get vaccinated can be noticed and the willingness ranges from 40% to 94% of the respondents, depending on the country [20,21]. Among others, a survey conducted in six European countries: Ireland, Norway, Switzerland, the Netherlands, Great Britain and Belgium, among pregnant or breastfeeding women indicates 40–50% willingness to undergo vaccination depending on the country [22].

Surprisingly, in the literature, there are downward trends observed as the pandemic continues and such a correlation has been observed, i.e., in the USA, where there was a decline of support toward vaccination from 76% in April 2020 to 56% in December 2020, despite numerous positive information regarding the effectiveness of the preparations [23]. In the present study, the opposite situation was noticed and the research showed that support for vaccination was increasing.

Over time, a reduction in the level of concern was observed regarding the lack of proper testing of the formulations and the way the products were transported and/or stored. A worrying phenomenon is an increase in the number of persons who are concerned about the effectiveness of vaccination even though many reports prove that vaccination reduces the risk of symptomatic COVID-19, hospitalisation including death caused by SARS-CoV-2 infection [24]. The above is very disturbing as it has been shown that the belief that the preparations were safe was the strongest predictor of the willingness to get vaccinated, more powerful than fear of getting ill [25]. 

According to this study, women manifest more favourable attitudes toward vaccination against COVID-19 than men. The above corresponds to current statistics regarding vaccination in Poland, according to which 65% of the vaccinated persons are women [26], however, the discussed results contradict the world reports that show that men are more enthusiastic toward vaccination than women [27]. The situation does not seem very different in the case of elderly people, persons with a higher level of education and those living in large cities. Persons who have received both mandatory and recommended vaccinations demonstrate more positive attitudes toward getting vaccinated and they are also convinced that vaccination against COVID-19 should be compulsory for everyone. Although persons who suffer from chronic conditions such as heart disease, lung disease, diabetes, etc., are much more likely to experience a severe course of the disease caused by COVID-19 infection, including death, they are not much more willing to get vaccinated, according to this study [28].

Approximately 20% of the respondents, in both stages of the study, were healthcare professionals. Their attitudes related to vaccination against COVID-19 are significantly different, according to this research. Out of 421 persons, as many as 54.9% have already been vaccinated. Every second person in the above-mentioned group of respondents expresses a desire to get vaccinated as soon as possible. Over 50% of healthcare professionals believe that vaccination should be mandatory for everyone. Those results are consistent with reports from other countries, according to which the majority of healthcare professionals are willing to get vaccinated, e.g., France (76.9%), Malta (61.8%) and Israel (71.8%) [29,30].

The very low level of willingness to get vaccinated with a vector vaccine, especially AstraZeneca, also requires a comment. The reason for the above might be the fact that during the period of the distribution of the survey, there were media reports that the preparation increases the risk of getting vein thrombosis [31]. Consequently, within a few days, vaccination with AstraZeneca was temporarily suspended, e.g., in Germany, Austria and Norway [31]. On the day before the finalisation of the collection of the data for this study, the European Medicines Agency (EMA) issued a statement regarding the above-mentioned formulation according to which the process of vaccination was to be continued and that there was no dependence between the risk of getting thrombosis and vaccination with AstraZeneca [32]. What is more, the approval of another type of vector vaccine, Johnson & Johnson, was published a week before the end of stage II of the study and the process of vaccination with the product has not started in Poland yet [9].

The mass media play a very important role in everyone’s life and it is the main source of information for millions of people about every aspect of life, every day. It has been confirmed by many studies that the media have a powerful influence on creating opinions and attitudes of people. It has also been demonstrated, inter alia, that frequent reading of media reports, browsing the Internet and becoming familiar with the information on COVID-19 caused the poor mental condition of individuals [33]. According to the literature on the subject, the media, including the Internet, are one of the most common sources of information regarding vaccination, which was also demonstrated in this study [34]. The strength of the media is their great accessibility, simplicity of use and high speed of obtaining information. However, a huge problem of the media is the large amount of fake news related to the effectiveness and safety of vaccination, the spread of conspiracy theories and questioning the existence of the pandemics or the need to get vaccinated which causes the escalation of anti-vaccination propaganda and hinders the efficient implementation of population vaccination. The authors of the study are aware of the limitations of the research caused by the method of data collection via an online survey. This method makes it impossible to estimate the information coverage of the survey and it is also very difficult to determine the percentage of uncompleted questionnaires at each stage of the research. The fact that the presented group of respondents is not representative of Polish society is also undoubtedly a limitation. The vast majority of women, persons with a higher level of education and younger people, i.e., <50 years old may have significantly affected the final results of the study. However, as it is well known, those are the individuals who are more likely to take part in any kind of survey distributed over the Internet [22,23,25]. It should be noted that, based on available information, this study is the first one conducted in Poland that compares the willingness of people to vaccinate against COVID-19 before and during the vaccination programme. The development of an effective COVID-19 vaccine in such a short time is a huge success for the scientific world. However, according to previous reports, the fact that there is a formulation is not a success and it is important to take steps to increase people’s awareness of vaccination and thus encourage them to get vaccinated [11].

To conclude, the correct implementation of the preventive vaccination programme in each country is currently one of the priorities of healthcare systems. Achieving an adequate number of vaccinated people would significantly reduce the spread of SARS-CoV-2 and it is an important element of the fight against the pandemic. Despite the ongoing population-based vaccination programme and the large number of campaigns to encourage people, the level of the willingness to get vaccinated remains at 50% which indicates low effectiveness of the promotional activities. The promotional campaign needs to be modified. For this purpose, the mass media can be used, especially the Internet and television which are, according to the respondents, the main source of information. As it was revealed in stages I and II of the study, nearly one in four respondents are unable to decide against vaccination or are willing to get vaccinated but not earlier than in a year. Those individuals should be the main target of pro-vaccination campaigns. During promotional activities, emphasis should be placed on increasing public awareness with the use of various reports that confirm the effectiveness and, most importantly, the safety of approved products because, as experience has shown, this is the main predictor of willingness to get vaccinated [24].

To determine the possibility of effective implementation of the vaccination programme in Poland, further observation and evaluation of social attitudes toward vaccination is necessary.

## 5. Conclusions

As the social vaccination promotional campaign continues, a slight increase in the willingness to get vaccinated against COVID-19 is observed. Women, persons living in large cities and those with a higher level of education demonstrate more favourable attitudes toward vaccination against COVID-19. It is worth noting that, despite the passage of time and increasing experience with new types of vaccines against COVID-19, the percentage of persons concerned about vaccine adverse events has not significantly decreased and, at the same time, the belief that the vaccines are ineffective has considerably increased. This demonstrates the limited effectiveness of the current information system regarding passing the knowledge on the safety and efficiency of vaccination and it indicates a necessity to modernise it as soon as possible. It is still necessary to observe the trends in the society to be able to respond to emerging incorrect information regarding vaccination and it is important to intensify the campaign to promote the national vaccination programme to achieve a satisfactory percentage of vaccinated persons and to guarantee herd immunity.

## Figures and Tables

**Table 1:** Characteristics of the study group with regard to the stages of the study.

Variable		Stage I (*n* = 463)	Stage II (*n* = 1559)	*p*
Age	18–29	183 (39.5%)	774 (49.7%)	<0.001
	30–39	131 (28.3%)	457 (29.3%)	
	40–59	127 (27.4%)	277 (17.8%)	
	≥60	22 (4.8%)	51 (3.3%)	
Gender	Male	114 (24.6%)	265 (17%)	0.002
	Female	349 (75.4%)	1294 (83%)	
Place of residence	Rural area	96 (20.7%)	340 (21.8%)	0.184
	City <50,000 residents	87 (18.8%)	264 (16.9%)	
	City of 50,000–250,000 residents	103 (22.3%)	291 (18.7%)	
	City >250,000 residents	177 (38.2%)	664 (42.6%)	
Level of education	Primary	1 (0.2%)	10 (0.6%)	0.267
	Lower secondary	5 (1%)	8 (0.5%)	
	Vocational	18 (3.9%)	40 (2.6%)	
	Secondary education	136 (29.5%)	462 (29.6%)	
	University degree	303 (65.4%)	1039 (66.7%)	
Relationship status	Married	212 (45.8%)	688 (44.1%)	0.845
	Civil partnership/Informal relationship	147 (31.8%)	504 (32.4%)	
	I am not in a relationship	104 (22.4%)	367 (23.5%)	
Healthcare professional	Yes	108 (23.3%)	313 (20.8%)	0.131
	No	355 (76.7%)	1246 (79.2%)	
COVID-19 diagnosis	In the course of the illness	5 (1%)	22 (1.4%)	0.472
	Convalescent	69 (15%)	265 (17%)	
	Has not been ill	389 (84%)	1271 (81.6%)	
Chronic conditions	Yes	-	529 (34%)	-
	No	-	1030 (66%)	
Person vaccinated against COVID-19	Yes	-	422 (27.3%)	-
	No	-	1033 (72.7%)	

**Table 2:** Comparison of attitudes and concerns related to vaccination against COVID-19 in both stages of the study.

Attitudes and Concerns to Cavvination against COVID-19		December 2020	March 2021	*p*
Willingness to vaccinate against COVID-19 *	Yes, as soon as possible	171 (36.9%)	507 (44.7%)	0.001
	Yes, but only in a few months	52 (11.2%)	66 (5.9%)	
	Yes, but in a year or more	12 (2.6%)	22 (1.5%)	
	No, but I might consider it in the future	81 (17.5%)	209 (18.5%)	
	No, never	113 (24.4%)	254 (22.4%)	
	I cannot make a decision	34 (7.3%)	77 (6.8%)	
Concerns over vaccination against COVID-19	Vaccine adverse event	240 (51.8%)	741 (47.5%)	0.265
	Lack of proper testing of vaccines	235 (50.8%)	593 (38.1%)	<0.001
	Improper transport/storage	122 (26.4%)	209 (13.4%)	<0.001
	Lack of effectiveness	91 (21.2%)	430 (27.6%)	0.022
	Other	13 (2.8%)	98 (6.3%)	0.015
	I have no concerns	57 (12.5%)	443 (28.4%)	<0.001
	The pandemic is a conspiracy	66 (14.3%)	149 (9.6%)	0.015
Past vaccinations	Yes, only mandatory	251 (52.2%)	920 (59%)	0.744
	Yes, mandatory and recommended	188 (40.6%)	572 (36.7%)	
	No	24 (5.2%)	67 (4.3%)	

* excluding persons who are already vaccinated.

**Table 3:** Attitudes toward vaccination against COVID-19 concerning socio-demographic variables, types of vaccination, past medical history including COVID-19.

Variable		Willingness to Get Vaccinated against COVID-19 * (* *n* = 1598) *n* (%)							Mandatory Vaccinations (*n* = 1559) *n* (%)			
		Yes, as Soon as Possible	Yes, but Only in a Few Months	Yes, but in a Year or More	No, but I Might Consider It in the Future	No, Never	I Cannot Make a Decision	*p*	Yes	No	I Do not Have an Opinion	*p*
Gender	Female	554 (43.9)	94 (7.5)	28 (2.2)	226 (17.9)	262 (20.7)	98 (7.8)	<0.001	529 (40.9)	518 (40.0)	247 (19.1)	<0.001
	Male	124 (37)	24 (7.2)	6 (1.8)	64 (19.1)	104 (31)	13 (3.9)		93 (35.2)	140 (53.0)	31 (11.7)	
Age	18–29	299 (37)	57 (7.05)	20 (2.5)	181 (22.4)	189 (23.4)	63 (7.8)	<0.001	275 (35.5)	364 (37.0)	135 (17.5)	0.004
	30–39	207 (46.6)	35 (7.9)	10 (2.3)	61 (13.8)	104 (23.4)	27 (6.0)		193 (42.3)	175 (38.4)	88 (19.3)	
	40–59	131 (47.0)	24 (8.6)	4 (1.4)	43 (15.4)	61 (21.9)	16 (5.8)		132 (47.7)	97 (35.0)	48 (17.3)	
	≥60	41 (63.1)	2 (3.1)	0 (0.0)	5 (7.7)	12 (18.4)	5 (7.7)		22 (43.1)	22 (43.1)	7 (13.8)	
Place of residence	Rural area	124 (33.9)	24 (6.6)	7 (2)	69 (18.9)	110 (30.0)	32 (8.7)	<0.001	114 (33.5)	165 (48.6)	61 (17.9)	0.004
	City <50,000 residents	100 (37.2)	23 (8.6)	4 (1.5)	54 (20.1)	63 (23.4)	25 (9.3)		104 (39.4)	106 (40.1)	54 (20.5)	
	City of 50,000–250,000 residents	121 (38.2)	24 (7.6)	8 (2.5)	59 (18.6)	85 (26.8)	20 (6.3)		104 (35.7)	136 (46.7)	51 (17.5)	
	City > 250,000 residents	333 (51.6)	47 (7.3)	15 (2.3)	108 (16.7)	108 (16.7)	34 (5.3)		300 (45.2)	251 (37.9)	112 (16.9)	
Education	Primary	4 (36.4)	3 (27.3)	0 (0.0)	1 (9.1)	2 (18.2)	1 (9.1)	<0.001	3 (30)	6 (60)	1 (10)	0.029
	Lower secondary	6 (46.2)	1 (7.7)	0 (0.0)	0 (0.0)	6 (46.2)	0 (0.0)		4 (50)	3 (37.5)	1 (12.5)	
	Vocational	13 (24.5)	1 (1.9)	1 (1.9)	6 (11.3)	28 (52.8)	4 (7.6)		9 (22.5)	21 (52.5)	10 (25)	
	Secondary education	201 (37.8)	31 (5.8)	10 (1.9)	101 (19.0)	147 (27.6)	42 (7.9)		167 (36.2)	221 (48)	73 (15.8)	
	University degree	454 (46.0)	82 (8.3)	23 (2.3)	182 (18.4)	183 (18.5)	64 (6.5)		439 (42.6)	407 (39.2)	193 (18.6)	
Relationship status	Married	325 (48.5)	51 (7.6)	13 (1.9)	105 (15.7)	139 (20.8)	37 (5.5)	0.003	307 (44.7)	255 (37.1)	125 (18.2)	0.005
	Civil partnership/Informal relationship	190 (35.5)	36 (6.7)	14 (2.6)	114 (21.3)	140 (26.2)	41 (7.7)		180 (35.7)	234 (46.4)	90 (17.9)	
	I am not in a relationship	163 (41.6)	31 (7.9)	7 (1.8)	71 (18.1)	87 (22.2)	33 (8.4)		135 (36.8)	169 (46.1)	63 (17.1)	
Healthcare professional	Yes	90 (47.4)	18 (9.5)	4 (2.1)	38 (20.0)	25 (13.2)	15 (7.9)	0.031	162 (51.8)	94 (30.0)	57 (18.2)	<0.001
	No	588 (41.8)	100 (7.1)	30 (2.1)	252 (17.9)	341 (24.2)	96 (6.8)		460 (37.0)	564 (45.3)	221 (17.8)	
COVID-19	In the course of the illness	15 (60.0)	2 (8.00)	0 (0.0)	2 (8.0)	5 (20.0)	1 (4.0)	0.056	10 (45.5)	8 (36.4)	4 (18.2)	0.517
	Convalescent	94 (38.2)	22 (8.9)	3 (1.2)	63 (25.6)	47 (19.1)	17 (6.9)		95 (35.9)	115 (43.4)	55 (20.8)	
	Has not been ill	569 (42.9)	94 (7.1)	31 (2.3)	225 (17.0)	314 (23.7)	93 (7.0)		517 (40.7)	535 (42.1)	219 (17.2)	
Past vaccinations (*n* = 1133)	Yes, only mandatory	308 (32.4)	59 (6.2)	18 (1.9)	214 (22.5)	275 (29.0)	76 (8.0)	<0.001	172 (24.6)	402 (57.6)	124 (17.8)	<0.001
	Yes, mandatory and recommended	357 (62.9)	54 (9.5)	16 (2.8)	67 (11.8)	42 (7.4)	32 (5.6)		185 (48.7)	56 (14.6)	139 (36.7)	
	No	13 (16.5)	5 (6.3)	0 (0.0)	9 (11.4)	49 (62.0)	3 (3.8)		13 (23.6)	38 (69.1)	4 (7.3)	
Chronic conditions (*n* = 1133)	Yes	191 (50.3)	19 (5.0)	8 (2.11)	60 (15.8)	77 (20.3)	25 (6.7)	0.155	239 (45.2)	197 (37.2)	93 (17.6)	
	No	315 (41.8)	47 (6.2)	14 (1.9)	149 (19.9)	176 (23.4)	52 (6.9)		383 (37.2)	461 (44.9)	185 (18.0)	

The results are presented as *n* and %. * excluding persons who are already vaccinated.

**Table 4:** List of concerns related to vaccination of persons who do not wish to get vaccinated.

Type of Concern	Stage I (*n* = 194) *n* (%)	Stage II (*n* = 463) *n* (%)
Vaccine adverse event	122 (62.9)	301 (65.1)
Lack of proper testing of vaccines	137 (70.6)	343 (74.1)
Improper transport/storage	42 (21.6)	65 (14.0)
Lack of effectiveness	59 (30.4)	208 (44.9)
Other	5 (2.3)	59 (12.7)
I have no concerns	2 (1)	25 (5.4)
The pandemic is a conspiracy	65 (33.5)	137 (29.6)

## Data Availability

The data presented in this study are available on request from the corresponding author.

## Supplementary Materials

The following is available online at https://www.mdpi.com/article/10.3390/vaccines9050503/s1. Table S1: The questionnaire designed by the author.
