# Supplementary material for: Attitudes toward Vaccination against COVID-19 in Poland. A Longitudinal Study Performed before and Two Months after the Commencement of the Population Vaccination Programme in Poland
Source: Vaccines (Basel). 2021 May 13;9(5):503. doi: 10.3390/vaccines9050503 (PMC8152483; doi:10.3390/vaccines9050503)
Supplement: Supplementary file 1 [file vaccines-09-00503-s001.zip › vaccines-1218148-supplementary.pdf]

Supplementary material - The questionnaire designed by the author

Dear All,

We would like to get your feedback regarding vaccination against COVID-19.

The survey is fully anonymous, voluntary and you can opt out of the survey at any stage with no need to disclose the reason.

The survey should not take more than 4 minutes.

1. Would you like to take part in the study? Yes/No
2. Have you been vaccinated against COVID-19 Yes/No
3. Gender: Female [ ] Male [ ]
4. Age: ..... years old
5. Place of residence:
   a) Rural area
   b) City <50,000 residents
   c) City of 50,000 - 250,000 residents
   d) City >250,000 residents

6. Education:
   a) Primary
   b) Lower secondary
   c) Vocational
   d) Secondary education
   e) University degree

7. What is your marital status?:
   a) I am not in a relationship/ I am divorced
   b) Civil partnership/ Informal relationship
   c) Married

8. Are you a healthcare professional?
   a) Yes
   b) No

9. If yes, what is your profession?
   a) Medical doctor
   b) Dentist
   c) Nurse and midwife
   d) Paramedic
   e) Laboratory diagnostician
   f) Technician
   g) Pharmacist
   h) Other

10. Do you suffer from any chronic diseases?

a) Yes
b) No

11. If yes, what kind? (you can choose more than one option)
    a) Cardiovascular diseases (e.g. hypertension, cardiac failure)
    b) Respiratory diseases (e.g. asthma, COPD)
    c) Neurological diseases (e.g. MS, epilepsy)
    d) Oncological diseases (e.g. breast cancer, colorectal cancer, etc.).
    e) Mental illnesses (e.g. depression, anxiety disorders, addictions)
    f) Skin diseases (e.g. psoriasis, AD)
    g) Endocrine disorders (e.g. thyroid, thyroid gland, diabetes)
    h) Other
12. Have you been diagnosed with COVID-19?
    a) Yes, I am in the course of the illness
    b) Yes, I am a convalescent
    c) No

13. Are you planning to get vaccinated against COVID-19?
    a) I am fully vaccinated
    b) Yes, as soon as possible
    c) Yes, but only in a few months (up to a year)
    d) Yes, but in a year or more
    e) I cannot make a decision
    f) No, but I might consider it in the future
    g) No, never
14. What are your concerns regarding vaccination? (you can choose more than one option)
    a) Adverse events after vaccination
    b) The vaccines have not been sufficiently tested
    c) The vaccines are not transported/stored in a proper way
    d) The vaccines might not be effective enough
    e) I have no concerns
    f) The pandemic is a conspiracy
    g) Other

15. Have you ever received any mandatory and/or recommended vaccines?

    a) Yes, only mandatory
    b) Yes, mandatory and recommended
    c) No

16. If you could choose a preparation, which product would you prefer to get vaccinated with?

    a) Pfizer BioNtech
    b) Moderna
    c) AstraZeneca
    d) Johnson&Johnson
    e) It does not matter to me

17. Do you think that vaccination against COVID-19 should be mandatory for everyone?

a) Yes
b) No
c) I do not have an opinion

18. What are your sources of information regarding vaccination against COVID-19? (you can choose more than one option)

a) The Internet
b) TV
c) Medical doctor
d) Healthcare professionals (other than a medical doctor)
e) Information leaflets
f) Friends, family (other than healthcare professionals)
g) Other

Table S1. Comparison of medical and non-medical professions concerning assessment of the concerns related to vaccination against COVID-19 for both stages of the study.

	Stage I of the study			Stage II of the study		
Variable	Medical profession (n= 108) n (%)	Non-medical profession (n=355) n(%)	p	Medical profession (n=313) n (%)	Non-medical profession (n=1246) n (%)	p
Vaccine adverse event	47 (43.5)	193 (54.4)	0.048	106 (33.9)	635 (51.0)	<0.001
Lack of proper testing of vaccines	47 (43.5)	188 (53.0)	0.081	76 (24.3)	517 (41.5)	<0.001
Improper transport/ storage	40 (37.7)	82 (23.1)	0.003	32 (10.2)	177 (14.2)	0.063
Lack of effectiveness	15 (13.9)	63 (23.4)	0.002	67 (21.4)	363 (29.1)	0.006
Other	2 (1.9)	11 (3.1)	0.478	21 (6.7)	77 (6.2)	0.731
I have no concerns	17 (15.8)	68 (19.2)	0.41	123 (39.3)	350 (28.9)	0.001
The pandemic is a conspiracy	4 (3.7)	62 (17.5)	<0.001	14 (4.5)	135 (10.8)	0.006
